# Exercise-Based Real-time Telerehabilitation for Older Adult Patients Recently Discharged After Transcatheter Aortic Valve Implantation: Mixed Methods Feasibility Study

**DOI:** 10.2196/34819

**Published:** 2022-04-26

**Authors:** Barbara Cristina Brocki, Jan Jesper Andreasen, Jens Aaroe, Jane Andreasen, Charlotte Brun Thorup

**Affiliations:** 1 Department of Physiotherapy and Occupational Therapy Aalborg University Hospital Aalborg Denmark; 2 Department of Cardiothoracic Surgery Aalborg University Hospital and Clinical Institute, Aalborg University Aalborg Denmark; 3 Department of Cardiology Aalborg University Hospital Aalborg Denmark; 4 Clinic of Anesthesiology, Child Disease, Circulation and Women, Clinical Nursing Research Unit Aalborg University Hospital Aalborg Denmark

**Keywords:** telerehabilitation, transcatheter aortic valve implantation, cardiac surgery, cardiac rehabilitation, exercise training, older adults, tablet

## Abstract

**Background:**

The use of telehealth technology to improve functional recovery following transcatheter aortic valve implantation (TAVI) has not been investigated.

**Objective:**

In this study, we aimed to examine the feasibility of exercise-based cardiac telerehabilitation after TAVI.

**Methods:**

This was a single-center, prospective, nonrandomized study using a mixed methods approach. Data collection included testing, researchers’ observations, logbooks, and individual patient interviews, which were analyzed using a content analysis approach. The intervention lasted 3 weeks and consisted of home-based web-based exercise training, an activity tracker, a TAVI information website, and 1 web-based session with a nurse.

**Results:**

Of the initially included 13 patients, 5 (40%) completed the study and were interviewed; the median age was 82 (range 74-84) years, and the sample comprised 3 men and 2 women. Easy access to supervised exercise training at home with real-time feedback and use of the activity tracker to count daily steps were emphasized by the patients who completed the intervention. Reasons for patients not completing the program included poor data coverage, participants’ limited information technology skills, and a lack of functionality in the systems used. No adverse events were reported.

**Conclusions:**

Exercise-based telerehabilitation for older people after TAVI, in the population as included in this study, and delivered as a web-based intervention, does not seem feasible, as 60% (8/13) of patients did not complete the study. Those completing the intervention highly appreciated the real-time feedback during the web-based training sessions. Future studies should address aspects that support retention rates and enhance patients’ information technology skills.

## Introduction

### Background

Aortic valve stenosis affects approximately 3% of patients aged ≥75 years. Untreated aortic stenosis (AS) leads to dizziness, fainting, dyspnea, chest pain, heart failure, and sudden cardiac death [1]. Transcatheter aortic valve implantation (TAVI) is increasingly being used as a procedure of choice for older adult patients with severe AS and high perioperative mortality risk [1,2]. The number of TAVI procedures is expected to increase in the coming years because of an aging population [3] and the positive short- and long-term results of the procedure [4]. Thus, TAVI has recently been recommended in patients who are aged >65 years and are at low and intermediate risk from surgical aortic valve replacement [4].

To date, no major guidelines recommend cardiac rehabilitation (CR) after TAVI [5], although emerging evidence suggests that CR is safe and has the potential to reduce mortality and improve exercise capacity and quality of life [6-9]. Participation in CR soon after TAVI may be of particular importance as sedentary behavior in this often frail population with multiple comorbidities is related to a higher risk of mortality and functional decline 1 year after the procedure [10]. In Denmark, less than 20% of patients are referred to and participate in CR following TAVI [11]. Several factors hinder patients’ participation in CR, including old age [12], lack of availability of municipality-based CR, lack of continuity between hospitals and local health centers where CR programs are performed, and lack of individualized rehabilitation [13].

### Cardiac Telerehabilitation in General

Telerehabilitation is defined as the use of information and communication technologies to support rehabilitation [14,15]. Cardiac telerehabilitation (CTR) has proven to be as effective in decreasing morbidity and mortality as center- and hospital-based CR programs [16,17]. In a recently published systematic review, CTR was found to be as cost-effective as traditional center-based approaches [18]. CTR may enhance attendance rates and long-term adherence to rehabilitation recommendations because it is performed in the participants’ own environment and can thereby be incorporated into their daily routines [19,20]. CTR often consists of digitally available cardiac-related patient information and the use of different devices (eg, activity trackers or weight scales) that collect and transfer data to a personal health record or digital platform [21,22], whereas others provide supervised exercise training [16,23]. Considering that the participation of older adult patients in center-based CR programs is poor [12,24], CTR may resolve barriers that hinder CR use and improve adherence to CR programs and sustainability of effects following the program [25].

### CTR Following TAVI

The effectiveness of CTR following TAVI has not yet been investigated, probably because the use of modern technology in the older adult population is still limited [26-28]. Hence, we developed a digital CTR program (TeleTAVI) based on four elements: (1) supervised home-based web-based exercise training, (2) an activity tracker, (3) a website containing disease-specific patient education and training videos, and (4) 1 web-based session with a nurse specializing in the care of patients undergoing TAVI. The development process was based on a participatory design [29], including individual patient interviews and workshops with patients, health professionals, researchers, and system developers [30]. The aim of this study was to investigate the feasibility and usability of a CTR program, named TeleTAVI, delivered via a tablet to an older adult population who had recently undergone TAVI surgery, with consideration given to the potential barriers in the use of technology for this particular population. We hypothesized that patients who undergo TAVI would be able to manage and use a tablet containing a TeleTAVI program at home and would be positive regarding the TeleTAVI content and approach.

## Methods

### Overview

A prospective nonrandomized, single-center study using a mixed methods approach was designed to investigate the feasibility of the TeleTAVI program and evaluate patient experiences with the program. In addition, we collected data on the running expenses of the program. Furthermore, this study was conducted to gather information about whether and how a future large-scale randomized controlled trial could be performed. The first author (BCB) was in charge of all procedures for recruitment and running the study, while the last author (CBT) performed patient interviews. The study was reported in accordance with the CONSORT (Consolidated Standards of Reporting Trials) extension for feasibility and pilot studies [31].

### Setting

Participants were recruited from the Department of Cardiology, Aalborg University Hospital, Denmark, between August 18 and September 22, 2020. The hospital performs 120 TAVI procedures each year. The Danish National Health Service provides tax-supported health care, including general CR, for all inhabitants, guaranteeing free access to family physicians and public hospitals.

### Ethics Approval

This study was approved by the Danish Data Protection Agency (registration 2020-054). The regional ethics committee stated that no approval was required for this study. Informed written consent was obtained from all participants before inclusion.

### Inclusion and Exclusion Criteria

Eligible participants were adults who planned for elective TAVI and were capable of reading and understanding Danish. Indications for TAVI in the present patient cohort were primarily high-risk, symptomatic AS, and or aged >80 years. The exclusion criteria were physical deficits that adversely influenced physical performance, decreased cognitive functioning, or TAVI performed as acute or subacute surgery.

### Surgery and Perioperative Management

TAVI was performed with local anesthesia and conscious sedation, with the insertion of a self-expandable aortic valve using a balloon catheter through a transfemoral incision. The choice of heart valve used (Edwards Sapien Ultra, Edwards Lifesciences) or Merill MyValve (Life Sciences Pvt Ltd) was made by the surgeon. After surgery, patients were transferred to the intensive care unit for observation and returned to the ward on the evening of the day of surgery or, at the latest, the next morning. When stable, patients were mobilized to walk on the day of surgery and were discharged within 2 or 3 postoperative days.

### Intervention

The technologies used for the pilot study are presented in Multimedia Appendix 1. The intervention was multimodal, lasted 3 weeks, and consisted of supervised web-based exercise training, patient support, the use of an activity tracker, and access to a project website.

### Technology and Management

The technologies were introduced during a home visit, 1 week after hospital discharge. A booklet containing written user instructions for each element of the intervention and a schedule of rehabilitation activities were provided to each patient before hospital discharge. The booklet was continuously adjusted during the study period according to patient feedback.

To deliver the video-training sessions at the hospital, we used a 49-in television monitor, a high-definition sound bar, and a Bluetooth headset to enable 2-way communication during each session.

### Tablet

All the participants received a tablet (iPad, Apple) along with a SIM card for data coverage. For the web-based training sessions, we used an encrypted videoconferencing system (Videosamtale) hosted by Aalborg University Hospital, that complies with the General Data Protection Regulation (GDPR) for European countries. During the home visit, patients were thoroughly introduced to how to connect to the web-based program and how to access the project website [32] for information and videos related to themes identified as important by patients who had previously undergone TAVI. For simplicity, the tablet setup only allowed the patients to use the TeleTAVI project’s website and an email program for assessing the link to the videoconferencing system.

### Activity Tracker

We used 2 different activity tracker models measuring step counts: Fitbit Charge 3 (Fitbit LLC) and Beurer AS 87 (Beurer Germany) to identify the most feasible activity tracker for use in a later extension of the program. The patients filed the daily number of steps in their training diaries, and we uploaded the data stored in each activity tracker after collecting the equipment at the patients’ homes.

### Exercise Training

Individualized web-based home exercise training followed the national recommendations for CR with a combination of aerobic and strength training twice weekly, with each session lasting 30 to 45 minutes [33]. The target intensity for the aerobic exercises was either a heart rate of 80 to 100 beats/min (patients wearing Fitbit) or a Borg CR10 dyspnea score from 3 to 5 [34] (patients wearing Beurer). The number of web-based sessions was set at 5. Patients were offered further sessions if they were able to attend. In addition, the patients were instructed to take a 30-minute walk daily with moderate intensity. Before hospital discharge, the patients were instructed to perform 3 exercises on alternate days until the home visit took place (home exercise program is provided in Multimedia Appendix 2).

### Follow-up Session With a Nurse

The 1 web-based session with a project nurse was established as a follow-up after hospital discharge. The topics during the sessions were based on patients’ perspectives on the development process of the TeleTAVI program [30]. Spouses participated in the sessions at their own discretion.

### Data Collection and Analysis

Eligible patients were approached for inclusion the day before their surgery.

### Assessments

Demographic and perioperative data were collected from patients’ medical records. The following assessments were performed the day before surgery to evaluate patients’ preoperative functional status and to target the exercise training program: 6-minute walk test [35]; 30 seconds-sit-to-stand test to assess functional lower extremity muscle strength [36]; 4-m walk test to assess gait speed. A gait speed <0.7 m/s is defined as frailty in TAVI [37]. Dominant hand grip strength was also assessed using the a digital hand dynamometer [36] and Mini Mental Scale Evaluation [38]. For health-related quality of life, we used HeartQol [39], which is a disease-specific questionnaire validated for patients who have undergone cardiac valve replacement surgery [39,40]. For frailty, we used the Tilburg Frailty Indicator, a validated self-administered instrument for assessing multidimensional frailty in older populations [41]. The number of steps was recorded and compared with those registered in the patients’ step diaries. Furthermore, we collected data on the number of home visits for technical support and telephone calls regarding difficulties in using the tablet and log-in procedure. Data were stored using the REDCap (Research Electronic Data Capture) electronic data capture tool (REDCap Consortium, Vanderbilt University Medical Center) hosted by the North Denmark Region.

### Field Notes and Logbooks

Field notes consisted of field observations and logbook registrations of each patient regarding their participation in the CTR program.

#### Patient Interviews

Individual interviews with patients completing the CTR program were performed to gain insight into their experiences of being part of the TeleTAVI program and the usability of technologies and devices. The interviews were based on a semistructured interview guide [42] (Multimedia Appendix 3) and lasted 30 to 90 minutes. All interviews were conducted in the patients’ homes at the end of the intervention, and partners were invited to participate. The interviews were digitally recorded and transcribed verbatim by a research assistant.

#### Estimated Costs

The running expenses for the program were estimated per patient completing the program and expressed as costs related to the equipment delivered to each patient at home (tablet, activity tracker, and home training equipment) and staff costs (transportation for home visits, running the web-based intervention, and information technology [IT] support).

#### Data Analysis

Descriptive statistics were used to describe the study population, and nonparametric statistics were used to analyze the differences between patients who completed the study and those who did not. A 2-sided *P* value <.05 was considered statistically significant. Owing to the small number of cases and subsequent skewed data, we have presented the results as median, minimum, and maximum, as well as numbers, frequencies, and percentages when appropriate. Analyses were performed using SPSS software (IBM Analytics). No formal sample size calculation was performed because of the explorative character of the study and because no efficacy testing was performed [43].

The first author (BCB) read all the observations and comments registered in the research diaries. Themes were identified according to the elements that comprised the intervention, and the findings were reviewed and discussed with the last author (CBT). The analysis of each individual interview was conducted as a deductive manifest content analysis with the aim of creating a condensation of meaning [44]. After familiarization with the text, the interviews were coded and abstracted into categories and subcategories, using the NVivo (QSR International) coding system [45]. Both authors reviewed the categories and analyzed them according to the different elements of the intervention. The results are presented as a joint display [46], that is, both quantitative and qualitative results are presented together, according to the source of data: patient citations from the interviews, logbooks, or field notes.

## Results

### Overview

**Figure 1:**
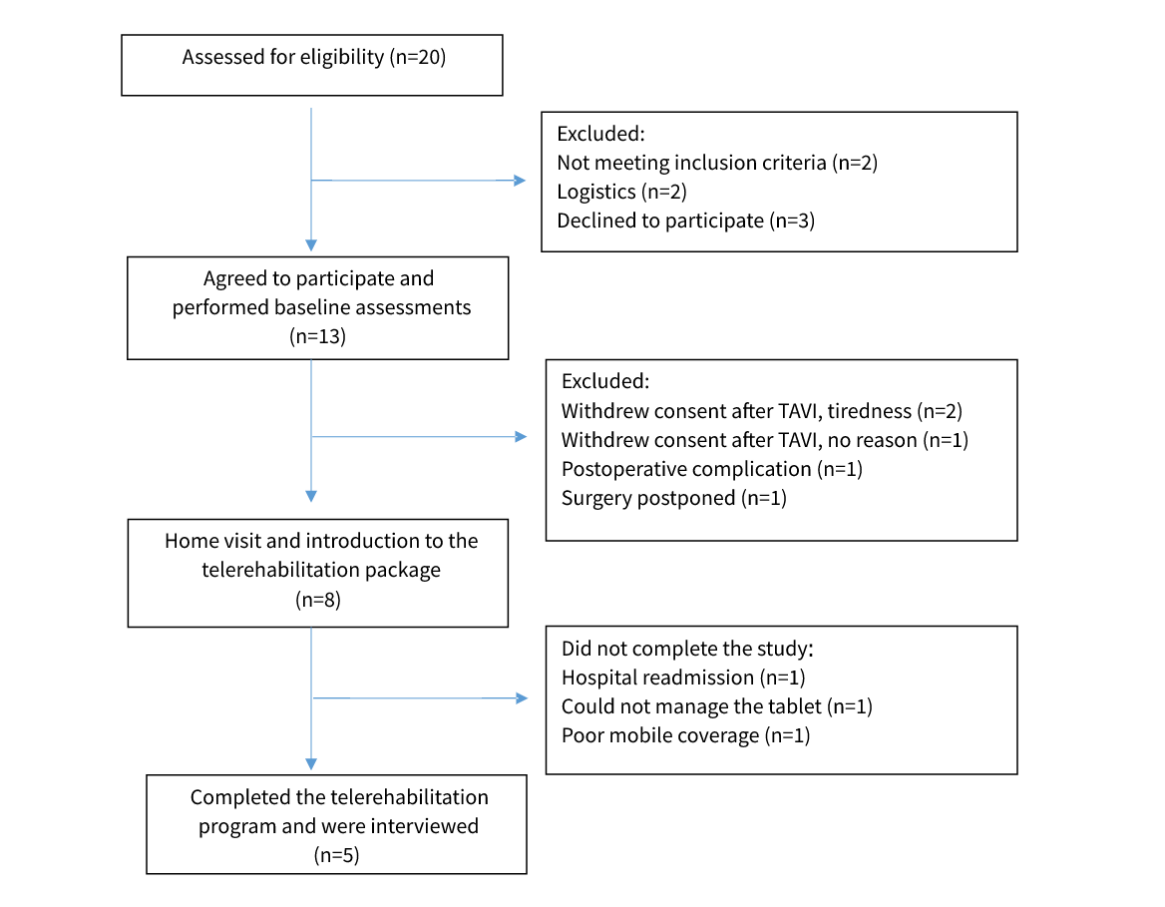
Study flowchart. TAVI: transcatheter aortic valve implantation.

In total, 20 consecutive patients admitted to Aalborg University Hospital for elective TAVI were assessed for eligibility; 13 patients with a median age of 83 years (range 74-87 years) agreed to participate and underwent baseline assessments. The median length of hospital stay was 3 days (range 3-30 days). Five patients (3 men and 2 women) completed the study. All had some experience with either the use of a computer or tablet, or they could get help from their relatives to manage the technology. Frailty was detected in a single patient completing the study, whereas 3 patients in the dropout group were categorized as frail (Table 1). The reasons for dropouts included tiredness after the surgery (n=2), hospital readmission (n=1), and poor mobile coverage (n=1; Figure 1, study flowchart). The first 3 patients included were introduced to the technology on the first postoperative day and reported that they were tired and could not concentrate on the technology at that time. Thus, the introduction of the technology was scheduled 1 week after hospital discharge.

The results and findings are presented as a joint display (Table 2) and summarized into the following categories: home-based rehabilitation, web-based exercise training, activity tracker, web-based session with the nurse, and website and technical issues. Each category was elaborated separately, and quotations from the interviews were provided to illustrate the findings.

**Table 1:** Demographics and surgical characteristics of participants.

Variables					Included (N=13)			Completed the study (n=5)			Did not complete the study (n=8)			*P* value^a^	
Age (years), median (range)					83 (74-87)			82 (74-84)			83 (75-87)			.35	
Gender (man), n (%)					8 (63)			3 (60)			5 (63)			.98	
BMI (kg/m^2^), median (range)					26 (23-30)			26 (23-27)			28 (24-30)			.22	
**Comorbidities, n (%)**															
	Arterial hypertension			8 (62)			3 (60)			5 (63)			.92		
	Ischemic heart disease			4 (27)			2 (40)			2 (25)			.57		
	Previous stroke			2 (15)			1 (20)			1 (13)			.83		
	Atrial fibrillation			3 (23)			2 (40)			1 (13)			.12		
	Diabetes mellitus			4 (27)			1 (20)			3 (37)			.67		
Left ventricular ejection fraction, median (range)					60 (40-60)			60 (40-60)			60 (45-60)			.82	
**NYHA,^b^ n (%)**															.28
	NYHA class II			8 (62)			4 (80)			4 (50)					
	NYHA class III			5 (38)			1 (20)			4 (50)					
**American Society of Anesthesiology Score, n (%)**															.83
	3			3 (23)			1 (20)			2 (25)					
	4			10 (77)			4 (80)			6 (75)					
Forced expiratory value first second, median (range)					77 (52-132)			61 (52-132)			80 (52-125)			.72	
Aortic peak gradient, median (range)					83 (50-140)			77 (50-140)			87 (55-105)			.43	
Hemoglobin, median (range)					8.2 (6.6-9.5)			8.5 (7.2-8.9)			8.2 (6.6-9.5)			.43	
Length of hospital stay,^c^ median (range)					3 (3-30)			3 (3-6)			3.5 (3-30)			.35	
**Physical functioning**															
	Walked distance (6-minute walk test; m), median (range)			400 (136-543)			460 (299-543)			391 (136-499)			.17		
	Walked distance % expected, median (range)			97 (36-143)			104 (63-143)			97 (36-113)			.52		
	Gait speed 4 m, median (range)			03.90 (02.98-10.20)			03.71 (03.15-04.26)			04.15 (02.98-10.20)			.28		
	Sit-to-Stand Test (30 seconds), median (range)			10 (6-16)			11 (8-15)			10 (6-16)^d^			.52		
	Hand strength % expected, median (range)			123 (82-162)			108 (84-162)			127 (82-160)			.99		
Mini Mental State Examination, median (range)					30 (28-30)			30 (29-30)			30 (28-30)			.77	
HeartQoL Quality of Life questionnaire, median (range)					0.79 (0.21- 2.14)			0.57 (0.29-2.14)			1.29 (0.21- 2.14)			.22	
**Sociodemographic, n (%)**															
	Living alone			3 (23)			1 (20)			2 (25)			—^e^		
	**Educational level**														.12
		Public school or short education	8 (61)			2 (40)			6 (75)						
		Medium education	3 (23)			0 (0)			3 (37)						
		Long education	2 (15)			1 (20)			1 (12)						
	**Information technology skills **														.06
		Novice	3 (23)			0 (0)			3 (37)						
		Acquainted with tablet or PC^f^	10 (77)			5 (100)			5 (62)						
**Tilburg Frailty Indicator (total score), median (range)**					3 (0-8)			2 (0-8)			1(0-8)			.51	
	Not frail, n (%)			9 (69)			4 (80)			5 (63)					
	Frail (≥5 points), n (%)			4 (31)			1 (20)			3 (38)					

^a^A *P* value <.05 is considered statistically significant.

^b^NYHA: New York Hear Academy Functional Classification.

^c^Includes operative day.

^d^n=7.

^e^Not available.

^f^Patient or next of kin.

**Table 2:** Joint display of results and findings summarized into categories according to the source of data.

Categories	Source of data	
	Logbooks	Field notes
Home-based rehabilitation	Home visits for technology introduction: n=8; lasted 1.5-2 hours each. Additional home visits for technical support: n=6. Transportation between the hospital and patient’s homes varied from 20 to 80 km.	Easier to establish a relationship during the home visit when patients had met the health professional during hospital stay. Easier for patients to follow the instructions when these were practical.
Web-based exercise training	The number of training sessions per participant varied from 2 (n=1) to 7 (n=1). The number of participants per session varied from 1 to 3. The sessions lasted 30-40 minutes each. Heart rate during the aerobic exercises varied from 70 to 90 beats per minute. For the CR10 dyspnea, the reported rating was 3-4.	Two spouses joined the training sessions. No adverse events occurred during the web-based training sessions. Giving individual guidance during web-based sessions was a challenge when ≥2 patients participated. An advantage to monitor the heart rate for targeting training intensity. Trying exercises and training equipment during the home visit supported individualization of exercises for the web-based sessions.
Activity tracker	Number of steps per day: 1.868 to 17.280; distance varied from 1.457 to 7.840 m Number of days the units were used: 7-28 days	Three patients returned their training diaries. There was concordance between patient registered data and the unit’s stored data. Only 1 user registered data for all days.
Web-based session with the nurse	Five sessions took place, lasting from 20 to 45 minutes each. One session was as a telephone call.	Internet-based face-to-face meeting was a positive experience and the issues discussed were mostly of practical nature.
Website	Log-in entry data were not collected.	The introduction to the use of the website took place as the last part of the home visit.
Technical issues	Telephone guidance to the log-in procedure given to 4 of 5 users, often for the first session. One participant needed telephone guidance for all the sessions.	External challenge: unstable or insufficient 4G net coverage; program or net outage. User-related challenge: Information technology novice in the use of a touch screen or email program; guidance for session log-in was often necessary; impaired vision or hearing. Functionality Tablet: customized for each user; relatively small screen size, when ≥2 users are connected at the same time; user forgets to charge the battery. Equipment: a 124.5-cm monitor facilitates viewing users logged in; a large screen enhances provision of individual guidance for the web-based training.

### Home-Based Rehabilitation

The home setting was practical, and patients felt privileged to participate. Meeting the same health professionals throughout the whole process facilitated continuity and was appreciated by the patients and health professionals involved. Meanwhile, the introduction to the technologies and provision of technical support were time-consuming for the health care professionals.

Field notes showed that the practical tasks learned during the home visit supported most patients in using the technology and joining the web-based sessions.

The interviews revealed that patients completing the program were positive about the TeleTAVI program and felt cared for instead of feeling lonely after hospital discharge. The home-based setting was perceived by the patients as practical and as an advantage as no transportation to a community center was necessary. The home-based setting was also especially valued owing to restrictions on social interaction during the COVID-19 pandemic:

Well, my goodness, you have not only received a new heart valve, you have received such an embrace of what you [red. health professionals] have given, to be able to feel good afterwards and beyond. I just feel it’s been so good. One is shown the way forward.

Patient, woman

It’s a good thing too because if people are debilitated and are in doubt about whether you can hold to such a training trip. You can just be at home, and then jump on. So, I, that’s for sure. This is fine, Corona [red COVID-19] or not.

Patient, man

### Web-Based Exercise Training

The number of training sessions per participant ranged from 2 (n=1) to 7 (n=1), and no adverse events occurred. The instruction on the exercises and training equipment during the home visit was helpful for the later individualization of exercises and was also valued by the patients. Targeting the training intensity was feasible regardless of the method used (heart rate or level of dyspnea). However, it was a challenge for the instructor to provide individual guidance when more than 2 patients were connected in the same session.

Patients described web-based exercise training as motivating and “real,” and there were several contributory factors. First, it was owing to the use of known exercises. Second, the patients could see the physiotherapist on the screen when receiving guidance on correct exercise performance, and they were able to exercise the whole body. Third, they felt committed to the web-based sessions, although such commitment could also be a barrier to performing the usual daily activities. Although one-on-one web-based training seemed to be the most efficient, voiced as “to see the instructor was the most important,*”* exercising in a group could also be motivating as it enhanced the feeling of not being alone:

I think it has been nice to have things shown. And I think it has been great to have the tablet to look at when we did the exercises. So, it was nice, also like today where you could correct me if it was wrong or it was right, right? So, I think it’s been fine.

Patient, woman

“We often said to each other” There are some muscles we do not use, we think “you do not need to do”, but when we have finished [red. training], there were some muscles we have used, which we do not usually use, so just like the arms all the way up and like that, that’s not how we are used to.

Spouse, woman

It [red. training] was on certain days, so I had to get it over, then I could give myself to do something else. I could not go out in the fields or anything else before it was over.

Patient, man

Well, I can tell you. When we stand and do it [red. training], I feel, well you’re in here in the living room, you are standing and directing and your friends there, they are standing here. This is how I feel, we’re a small bunch of people.

Patient, woman

### Activity Tracker

There was a large variation in the number of steps taken per day among the patients, varying from 1868 to 17,280. The patients perceived wearing an activity tracker as a way to verify the usual number of daily steps taken. Expressions such as *“*all steps count*”* often occurred throughout the interviews when patients described positive experiences while wearing the device, which could be a motivation to increase the daily number of steps. Others did not wear the device throughout the intervention period, either because they were reassured that their usual daily steps exceeded the recommendations or because they did not understand how to manage the device:

Well, it was motivating because that, then I reach the 1700 [steps] here, you know, well, then I’ll take a walk up in the woods and reach 2.000

Patient, man

It has not worked, just lying on the table there, with power on. I thought it was missing power, but then you said I should wear it in my wrist, and then the shit worked. Then I went on the big walk, to get many steps.

Patient, man

### Web-Based Session With the Nurse

In total, 5 web-based sessions were conducted. The issues discussed were mostly of a practical nature, such as medication, pain, and sleeping. The project nurse experienced the internet-based face-to-face conversation with the patients as positive as their body language was visible, which indicated the patients’ actual well-being. Although most of them could not recall the specific issues discussed, the patients and their spouses appreciated the provision of follow-up after hospital discharge:

Can well remember that we should get ready for the conversation. I think it gives a bit of reassurance, there is someone who is interested in you, right?

Spouse, woman

### Website

Overall, the project website was only occasionally used by the patients, mostly because they forgot that they could access it. When it was used, patients, and eventually their spouses, appreciated watching videos in which other patients talked about their own course of disease, treatment, and recovery. The patients were not interested in viewing videos with self-training information:

I watched patient and relatives’ videos, that is, the different ones telling about how they have experienced it. The videos were very, very good, mostly listened to the videos, not read that much.

Patient, woman

### Technical Issues

Challenges regarding the use of this technology were experienced by both patients and health care professionals. These were categorized as external or user or functionality related.

The main external challenge was unstable or insufficient 4G data coverage, mostly in less-populated areas, which could often be solved by connecting the tablet to the users’ Wi-Fi when available. One dropout was owing to unstable data coverage.

User-related challenges were associated with a lack of prior experience with web-based communication platforms, such as handling emails or dealing with a touch screen, and this lack often required IT support, which was provided by telephone. Customization of the tablet was provided when necessary, for instance, by adjusting the period for screen touch. Patients expressed different ways of managing challenges with the use of a tablet, ranging from confidence to a lack of faith in their own ability. One patient expressed that he had no interest in the use of digital technology and left such issues to his spouse. Regardless of the individual approach taken, patients managed to use the tablet to participate in web-based training sessions:

I am not used to using a tablet. I have a computer that I always use. So that way, I’m used to using technology, but I’ve never used a tablet before.

Patient, man

I totally get [goose] bumps when I think about, no, you have to, can you, you cannot figure it out.

Patient, woman

Challenges related to tablet functionality were also identified. The main challenge for the patients was related to the tablet’s relatively small screen size and visual deficits as it was important to be able to see the instructor’s complete body so that they could better follow the exercises:

If there were many [participants], then the pictures got small, and then you have to get closer. It would be better if there was a big picture of you [instructor], and small of the others.

Patient, man

For the health care professionals, instructing the patients in the TeleTAVI during the home visits took 90 to 120 minutes, which meant that it was a time-consuming task and one that continued as they had to instruct and guide the patients afterward for logging into the training sessions.

### Estimated Costs

The estimated running cost for the program was US $ 1.467 per patient who completed the study. This included US $840 for equipment delivered to each patient and US $627 for staff costs.

## Discussion

### Principal Findings

Exercise-based telerehabilitation for the elderly after TAVI in the population as included in this study, and delivered as a web-based intervention, does not seem feasible as 60% (8/13) included patients did not complete the study. Barriers negatively influencing adherence to the program included poor data coverage, participants’ limited IT skills, and functionality of the systems used. Meanwhile, qualitative findings suggest that the TeleTAVI program supported personalized, tailored training interventions in patients completing the program. The home-based web-based delivery form of the exercise training sessions was appreciated by the patients because there was no need for transportation, and they felt that they exercised their whole body while receiving real-time feedback. However, the program was time-consuming for the health care professionals as a great deal of time was used for transportation, home instruction, and IT support. No adverse events were reported. Aspects that support retainment rates and enhance patients’ IT skills need to be further addressed before the program can be used on a larger scale, such as in a randomized controlled setting, as intended.

### Comparison With Prior Work

The findings from this first study on TAVI CTR are in line with existing knowledge of the use of CTR in patients with other cardiac conditions. In particular, easy access to exercise training without the need for transportation to a rehabilitation center is a well-described advantage that promotes patient engagement and adherence [24,47]. Exercise supervision is a key element in center-based CR to individualize exercises and provide sufficient training load to achieve gain in cardiorespiratory fitness [33]. In this study, we found that virtual feedback allowed for individualization during the training sessions, whereas the provision of exercise equipment facilitated patients to reach a proper training load. This was facilitated by face-to-face introduction to the exercises during the introductory home visit. These elements were also voiced as being important by the participating patients and their spouses, possibly supporting their adherence to the program. Furthermore, the use of adequate equipment for video-training delivery at the hospital facility was vital for enabling two-way communication during each session.

We were particularly challenged as many patients did not complete our study because they could not manage the technology or because of technical issues. First, in the short study period, we experienced outages in both the broadband connection and the video conferencing app. Stable internet connectivity was the premise for the use of the videoconferencing system. Even though the tablet had a 4G SIM card, we still experienced unstable data coverage in both rural and urban areas, a reason for the 2 patient withdrawals. If required and available, we connected the tablets to the patients’ own Wi-Fi to ensure proper running of the videoconferencing system and enhance program compliance. To date, many homes do not have internet. In 2019, up to 10% of Danish citizens reported not having broadband at home, particularly older adults aged 75 to 89 years, of whom 29% had never previously accessed the internet [48]. This may pose a challenge for future CTR telerehabilitation delivery, particularly in the elderly population. Second, according to the initial study protocol, we introduced patients to the technology during their hospital stay, which was probably not the best introduction time for new technology in this older population. Consequently, we adapted the protocol and introduced the technology during the home visit 1 week after hospital discharge and had no further patient withdrawals for this reason. Finally, the setup for the intervention was time-consuming for the health care professionals as a great deal of time was spent on introduction to the telerehabilitation packet, IT support, and transportation. This may also be a barrier to future implementation of CTR after TAVI.

### Future Directions

Findings from our feasibility study indicate that the use of telerehabilitation technology in older persons who have undergone TAVI, although challenging, is also promising as many patients are acquainted with the use of smartphones and tablets, and patients completing the program appreciated the home-based web-based setting. Therefore, we recommend changes in future TAVI-CTR interventions. First, extension of the program to 12 weeks post-TAVI will follow current guidelines for the duration of CR [33,49]. Furthermore, a longer intervention period may also facilitate patients to get more acquainted with the technology with additional less cost to the program in the long term. Second, the provision of remote IT support may help patients in using the tablet properly. Third, the use of a wireless platform for automatic uploading and collection of data on daily steps should be considered, conditional of complying with the GDPR regulations [22]. Devices with commercial applications that automatically upload to a tablet and store patients’ data on daily number of steps may not comply with GDPR regulations for data safety and privacy in research [50], although it poses no concern when used privately by patients. Fourth, the ownership of a smartphone [28] and digital access to the internet may be used as proxies for screening older patients for CTR. Finally, a reduction in the number of functions in a CTR program might enhance the willingness to participate in CTR and thus enhance retention rates.

With as few as 10% to 20% of patients attending CR after TAVI [11,23], delivery models that are alternatives to the established center-based CR still need to be developed and tested to enhance patient uptake to rehabilitation after surgery, as well as to establish evidence on the effect of CR following TAVI. In this context, CTR may be a cost-effective alternative to add-on interventions [18]. However, it is also important to bear in mind that patients who undergo TAVI are often octogenarians and frail [6,51], which may have influenced patient withdrawal in our study.

### Strengths and Limitations

As this was a single-center trial with no control group, our study has limited generalizability. In addition, we included only a small number of participants owing to the study’s proof-of-concept nature [43] with a limited inclusion period. However, it is a strength that we screened all the patients scheduled for TAVI in our hospital, which was similar to the feasibility randomized study performed by Rogers et al [8]. Apart from the walked distance, the age of the participants in our study and several clinical features, such as the presence of comorbidities, ejection fraction, and NYHA classification were similar to those in studies investigating the effect of CR following TAVI [8,51-54].

### Conclusions

In conclusion, we found that exercise-based telerehabilitation in older adult patients after TAVI, in the population as included to this study, delivered as a web-based intervention, does not seem feasible, as 60% (8/13) of the included patients did not complete the intervention. Conversely, we found several promising aspects favoring the web-based setting as real-time feedback during home training was highly appreciated by those who completed the intervention. Aspects that support retainment rates and enhance patients’ IT skills need to be further addressed before the program can be used on a larger scale, as intended, in the form of a randomized controlled trial.

## Appendix

Multimedia Appendix 1 Technologies.

Multimedia Appendix 2 Home exercise program.

Multimedia Appendix 3 Interview guide.

## Abbreviations

CONSORT: Consolidated Standards of Reporting Trials
CR: cardiac rehabilitation
CTR: cardiac telerehabilitation
GDPR: General Data Protection Regulation
IT: information technology
REDCap: Research Electronic Data Capture
TAVI: transcatheter aortic valve implantation
