# Supplementary material for: Exercise-Based Real-time Telerehabilitation for Older Adult Patients Recently Discharged After Transcatheter Aortic Valve Implantation: Mixed Methods Feasibility Study
Source: JMIR Rehabil Assist Technol. 2022 Apr 26;9(2):e34819. doi: 10.2196/34819 (PMC9092235; doi:10.2196/34819)
Supplement: Multimedia Appendix 1 [file rehab_v9i2e34819_app1.pdf]

Technology	Aims and content
Tablet<br>(Apple iPad, Wi-Fi, 10.2”, 4G)	• Outlook mail program for online exercise training and online communication with project personnel via a video conferencing system “Videosamtale application” installed in the unit. The system allowed for the physiotherapist to see all patients on the same screen and to communicate with them. Also, patients could see and hear the physiotherapist at the hospital, and see themselves and other patients on their own screen as well as hear all conversations.<br>• Access to the project´s website
Website<br>https://aalborguh.rn.dk/teletavi	TAVI website hosted by the North Jutland region and installed in the iPad containing:<br>• Text information on issues concerning treatment, lifestyle, medicine<br>• Videos with training programs<br>• Videos with patients’ experiences and information from health professionals
Activity tracker<br>(Fitbit charge 3 or Beurer AS 87)	• For daily step counts; worn on the wrist during daytime; only one of the two devices was delivered to a participant<br>• For Fitbit Charge 3: to monitor heart rate during the supervised training sessions<br>• The participants registered number of daily steps manually in a training diary. Data was also stored in the devices and were subsequently transferred to a database
Booklet in paper form	• Containing schedule for home visit, for online training and for self- training, when to charge the iPad and the activity tracker<br>• Patient information on how to: use a tablet; access to Outlook app and website; use the activity tracker<br>• Description of home exercises, also illustrated by pictures
Training equipment - participants	• Step bench, training mat, exercise elastic band, dumb bells (1 kg, 2 kg, 3 kg)<br>• Delivered at the introductory home visit and used for the online training sessions
Technology for the online sessions	• 49” TV monitor<br>• Poly Polycom Studio Audio/Video USB Soundbar<br>• Plantronics Voyager Focus UC MS bluetooth headset
