# Supplementary material for: Exercise-Based Real-time Telerehabilitation for Older Adult Patients Recently Discharged After Transcatheter Aortic Valve Implantation: Mixed Methods Feasibility Study
Source: JMIR Rehabil Assist Technol. 2022 Apr 26;9(2):e34819. doi: 10.2196/34819 (PMC9092235; doi:10.2196/34819)
Supplement: Multimedia Appendix 2 [file rehab_v9i2e34819_app2.pdf]

AALBORG UNIVERSITETSHOSPITAL
– i gode hænder

Sådan træner du efter en stentklap operation

Med tiden bliver kroppen mere skrøbelig, og samtidigt taber du gradvis din muskelmasse og muskelstyrke. Derfor er det vigtigt at vedligeholde dine kropsmuskler, så du fx kan blive ved med at gå sikkert op og ned ad trapper.

Her er nogle øvelser, du kan lave på egen hånd, efter at du er kommet hjem fra hospitalet. Du vil blive instrueret i øvelserne af en fysioterapeut, imens du er indlagt. Du vil også få udleveret de nødvendige træningsredskaber.

Nogle øvelser har flere sværhedsgrader. Sammen med fysioterapeuten, finder I frem til det niveau for øvelse, der bedst passer dig lige nu.

Træn 2-3 gange om ugen

Du skal arbejde hen imod at lave 3 x 15 gentagelser af hver øvelse 2-3 gange om ugen. Husk at holde cirka 2 minutters pause efter hvert sæt øvelse.

KONTAKT OG MERE VIDEN

Har du spørgsmål er du velkommen til at kontakte os.

Kontakt
Fysio- og Ergoterapiafdelingen
Tlf. 97 66 42 10

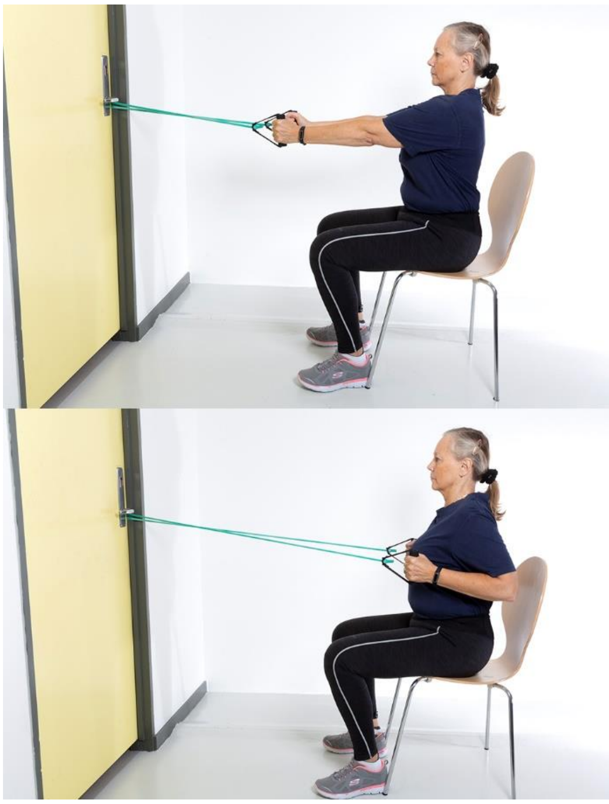

ØVELSE 1 - RONING

- Spænd elastikken fast bag en dørhåndtag.
- Sid på stolen med ret ryk, tag fat i elastikken og træk skulderbladene sammen.
- Træk langsomt hænderne mod brystet.
- Hold spændingen i ca. 3-5 sekunder.
- Stræk armene langsomt igen.

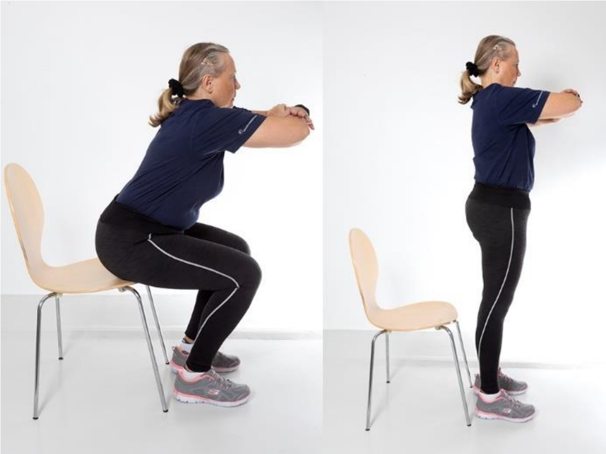

ØVELSE 2A – REJSE OG SÆTTE SIG

- Stå med ret ryg og armene over kors foran brystet.
- Sæt dig langsomt og kontrolleret ned.
- Bevæg kroppen fremad, imens du rejser dig op til stående igen.

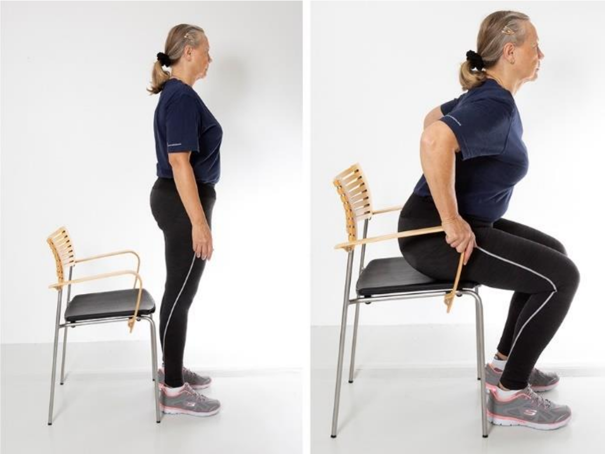

ØVELSE 2B - REJSE OG SÆTTE SIG VED BRUG AF STOLENS ARMLÆN

- Stå med ret ryg.
- Sæt dig langsomt og kontrolleret ned, imens du tager fat i stolens ryglæn.
- Bevæg kroppen fremad, imens du rejser dig op til stående igen.
- Ved behov, støt dig til stolens armlæn, når du rejser dig.

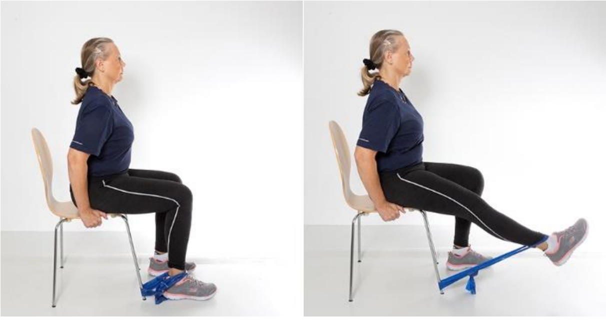

ØVELSE 2C – STYRKNING AF LÅRMUSKLER, HVIS DU IKKE KAN REJSE DIG FRA EN STOL

- Sæt elastikken stramt om anklerne.
- Sid på stolen med ret ryg, hold ved stolens sæde.
- Stræk knæet ud, hold stillingen i 3-5 sekunder inden du sætter foden tilbage.
- Lav 3 x 10 gentagelser.
- Gentag øvelsen med det modsatte ben.

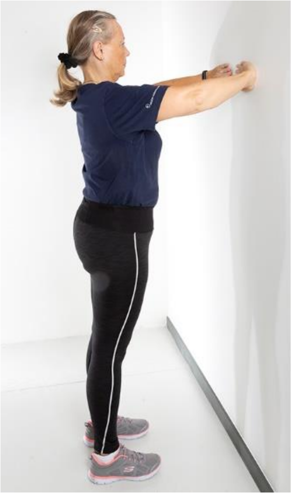
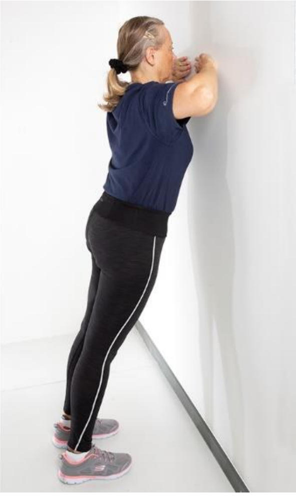

ØVELSE 3A – STABILITET I SKULDRE OG OVERKROP

- Stå med ret krop og hænderne placeret som på billedet.
- Træk skulderbladene sammen.
- Bøj armene langsomt uden at slippe skulderbladenes stilling.
- Stræk armene langsomt ud igen.

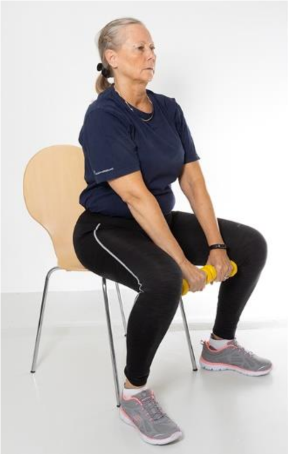
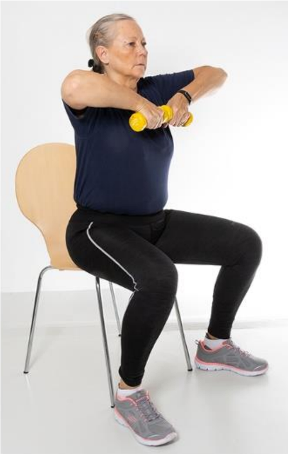

ØVELSE 3B – STABILITET I SKULDRE OG OVERKROP

- Hold en håndvægt i hver hånd.
- Ret ryggen og træk skulderbladene sammen.
- Løft håndvægte til brystet som på billedet.
- Hold stillingen i 3-5 sekunder og sænk håndvægte langsomt ned igen.

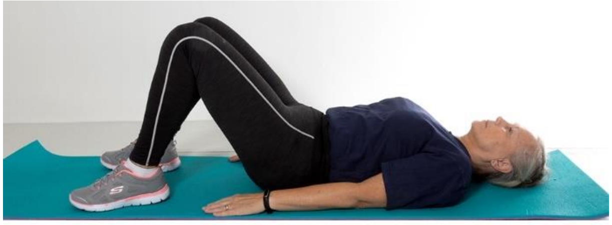
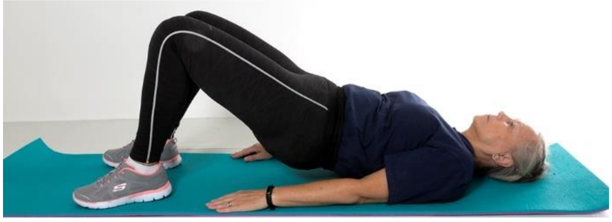

ØVELSE 4 – BÆKKENLØFT

- Lig på ryggen med bøjede knæ.
- Klem balderne sammen.
- Løft bækkenet og den nederste del af ryggen fra underlaget.
- Hold stillingen i 3-5 sekunder og sænk så bagdelen langsomt ned igen.

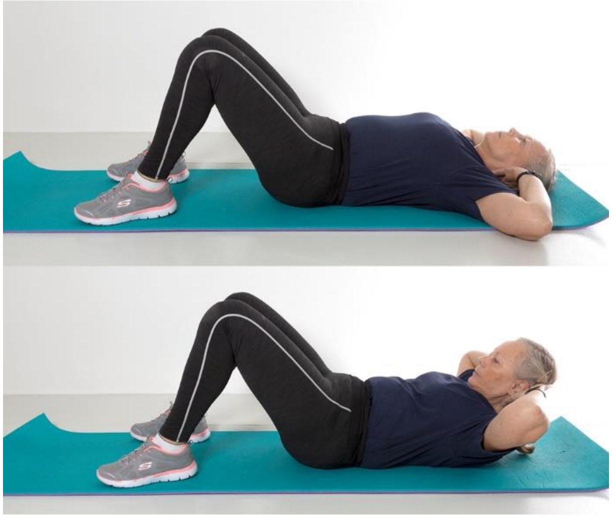

ØVELSE 5 – MAVEØVELSE

- Fold hænderne bag nakken.
- Pres hægen ind mod brystet samtidigt med at du presser lænderyggen ned i madrassen.
- Løft hovedet og skuldrene fri fra underlaget
- Hold stillingen i 3-5 sekunder.
- Sænk hovedet langsomt tilbage til udgangsstillingen og hold spændingen i mavemusklerne, indtil skuldrene hviler i madrassen.
- Obs: hold hægen ind mod brystet i hele bevægelsen.

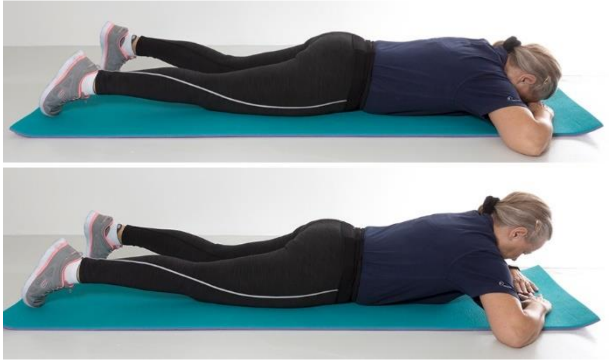

ØVELSE 6 – RYGØVELSE

- Lig på maven.
- Flyt armene frem, så din pande hviler på dine fingre.
- Løft hovedet fra underlaget, hold stillingen i 3-5 sekunder.
- Sænk hovedet, så din pande hviler på fingrene igen.
