# Supplementary material for: Exercise-Based Real-time Telerehabilitation for Older Adult Patients Recently Discharged After Transcatheter Aortic Valve Implantation: Mixed Methods Feasibility Study
Source: JMIR Rehabil Assist Technol. 2022 Apr 26;9(2):e34819. doi: 10.2196/34819 (PMC9092235; doi:10.2196/34819)
Supplement: Multimedia Appendix 3 [file rehab_v9i2e34819_app3.pdf]

Preparation and preliminary questions
Thank you for participating in this interview (repeat that it is voluntary, obtain signed consent, provide a copy to the participant).
I will ask you a series of questions. Try to put as many of your thoughts and considerations into words.

Themes	Main questions	Sub questions
Situation after hospital discharge	Tell me about the information you got on the project before you were discharged from the hospital.<br>How did you experience the time after you came home from the hospital?<br>Have you participated in other types of training?	Was the information easy to understand/to manage? Explain<br>Did you feel ready to come home then?<br><br>Training in elderly activity center/ municipality
Training and exercises	What has the home training meant to you?<br>Does this way of training motivate you?<br>Was the training program on paper easy to follow?	Was it practical, easy, difficult, unmanageable? Explain<br>How did you experience training with the step bench, training mat, dumb bells?<br>Was the program on paper helpful? How?
Tablet (instruction and information)	How did you experience using the tablet?<br>How did you experience the training via tablet?<br>What do you think of the information on the website?<br>What did the conversation with the nurse mean to you?<br>Does using the tablet motivate you?	Was it easy, difficult, practical, or was print too small?<br>How much have you used the tablet? Watched the videos, read the material?<br>Have you used the tablet to show others what the surgery was about?<br>(social relations, manageable, comprehensible, meaningful)<br>Have you used a tablet before? Do you use PC and email in everyday life?
Activity tracker	What did you think of the activity tracker?<br>What has it meant to you to use the activity tracker?<br>Does using the activity tracker motivate you?	Easy to wear/use/cumbersome, bother your wrist? Explain<br>Have you become more motivated to take a walk?
Overall	Can you briefly tell us what it has meant to you to train online and interact with us at the hospital in this way?	
Improvements	Is there anything you have been missing? (information?)<br>What could we have done differently?<br>What do you think could make others participate in training via tablet in the future?<br>What do you think could be a barrier for others to participate in this form of training?	

Debriefing: I have no more questions. Do you have more you´d like to say, ask for, or add before we end? Thanks for your participation
